# Potent neutralizing monoclonal antibodies against Ebola virus infection

**DOI:** 10.1038/srep25856

**Published:** 2016-05-16

**Authors:** Qi Zhang, Miao Gui, Xuefeng Niu, Shihua He, Ruoke Wang, Yupeng Feng, Andrea Kroeker, Yanan Zuo, Hua Wang, Ying Wang, Jiade Li, Chufang Li, Yi Shi, Xuanling Shi, George F. Gao, Ye Xiang, Xiangguo Qiu, Ling Chen, Linqi Zhang

**Affiliations:** 1 Comprehensive AIDS Research Center, and Collaborative Innovation Center for Diagnosis and Treatment of Infectious Diseases, School of Medicine, Tsinghua University, Beijing 100084, China; 2 Beijing Advanced Innovation Center for Structure Biology, and Collaborative Innovation Center for Diagnosis and Treatment of Infectious Diseases, School of Medicine, Tsinghua University, Beijing 100084, China; 3 State Key Laboratory of Respiratory Disease, First Affiliated Hospital of Guangzhou Medical University, Guangzhou 510230, China; 4 Special Pathogens Program, National Microbiology Laboratory, Public Health Agency of Canada, Winnipeg, Manitoba, R3E 3R2 Canada; 5 Department of Medical Microbiology, University of Manitoba, Winnipeg, Manitoba, R3E 0J9 Canada; 6 Guangzhou Institute of Biomedicine and Health, Chinese Academy of Sciences, Guangzhou, 510530, China; 7 CAS Key Laboratory of Pathogenic Microbiology and Immunology, Institute of Microbiology and Research Network of Immunity and Health, and Beijing Institutes of Life Science, Chinese Academy of Sciences, Beijing 100101, China

## Abstract

Ebola virus infections cause a deadly hemorrhagic disease for which no vaccines or therapeutics has received regulatory approval. Here we show isolation of three (Q206, Q314 and Q411) neutralizing monoclonal antibodies (mAbs) against the surface glycoprotein (GP) of Ebola virus identified in West Africa in 2014 through sequential immunization of Chinese rhesus macaques and antigen-specific single B cell sorting. These mAbs demonstrated potent neutralizing activities against both pseudo and live Ebola virus independent of complement. Biochemical, single particle EM, and mutagenesis analysis suggested Q206 and Q411 recognized novel epitopes in the head while Q314 targeted the glycan cap in the GP1 subunit. Q206 and Q411 appeared to influence GP binding to its receptor NPC1. Treatment with these mAbs provided partial but significant protection against disease in a mouse model of Ebola virus infection. These novel mAbs could serve as promising candidates for prophylactic and therapeutic interventions against Ebola virus infection.

Ebolavirus is the etiologic agent of lethal hemorrhagic fever in humans and nonhuman primates. Since its discovery in 1976, Ebolavirus has caused frequent outbreaks across Central Africa with exceedingly high mortality rate up to 90%^1^,^2^. While the natural host is currently unknown, Ebolavirus has been exploring more species as hosts across broader geographic frontiers^3^,^4^,^5^. Most notable was the unprecedented outbreak in West Africa in 2014 where the number of affected people has surpassed all the previously recorded cases combined^1^, raising the concerns about its pandemic potential and posing a serious threat to global health. Genetically, Ebolavirus isolated so far represents a divergent and evolving cluster and can be broadly classified into 5 species: Zaire (EBOV), Sudan (SUDV), Bundibugyo (BDBV), Reston (RESTV), and Tai Forest (TAFV) viruses^6^. Multiple lineages of EBOV were identified with increasing phylogenetic and genetic diversity in the 2014 outbreak in West Africa^7^,^8^,^9^,^10^,^11^,^12^. While it is uncertain whether the observed genetic diversity is the cause or the consequence of the current epidemic, the high mortality rate associated with rapid human to human transmission strongly argue for more effective therapeutics and vaccines against EBOV infection.

The surface glycoprotein (GP) of EBOV mediates viral entry and serves as the main target for antibody-based therapy and vaccination. Once translated inside the target cell, GP is cleaved by furin to yield disulphide-linked GP1 and GP2 subunits that further assemble into metastable trimers^13^. The GP1 subunit is responsible for cellular attachment and binding to the receptor Niemann Pick C1 (NPC1), whereas GP2 mediates fusion of the viral and cellular membranes^13^. Three GP1 subunits forms a chalice consisting of the receptor binding domains (RBD), the glycan caps and heavily glycosylated mucin-like domains (MLD)^14^. The RBDs are sequestered in the chalice bowl while the glycan caps and MLDs are projected at the rim of chalice to prevent immune recognition^14^. GP2 wraps around GP1 to forms the base of the chalice containing the fusion loop as well as N- and C-terminal heptad repeat regions critical for viral fusion^14^. During viral entry, GP undergoes proteolytic cleavage by endosomal cathepsin proteases to generate cleaved GP (GPcl) devoid of the glycan cap and MLD and exposes additional surface residues required for NPC1 binding^15^. In addition, the unedited GP gene encodes for soluble GP (sGP) which includes the glycan cap but lacks the MLD and GP2^13^. It has been shown that sGP is produced in larger quantity than GP during replication and may therefore act as a decoy by binding to neutralizing antibodies^13^. Such complexity in GP structure and insidious tactics to evade and distract immune recognition may partially explain why majority of infected individuals succumbed to diseases and failed to generate strong neutralizing antibody during natural infection^16^,^17^.

Recently, significant progress has been made in antibody therapy and vaccines against EBOV although no product has yet to receive regulatory approval^18^,^19^,^20^,^21^,^22^,^23^,^24^,^25^,^26^. In particular, monoclonal antibody (mAb) cocktails instead of single mAb conferred protection to nonhuman primates when passively administered after otherwise lethal viral challenge^21^,^22^,^24^,^25^,^26^,^27^. The most potent and successful cocktails include MB-003 consisting of human or human-mouse chimeric mAbs c13C6, h13F6 and c6D8^22^,^25^, ZMAb consisting of murine mAbs m1H3, m2G4 and m4G7^23^, and ZMapp consisting of human-mouse chimeric mAbs c13C6, c2G4 and c4G7^24^. Most strikingly, ZMapp demonstrated protection effect in nonhuman primates when administered as late as 5 days post infection and used on a compassionate basis in humans during the 2014 outbreak^24^,^28^. Epitope mapping of these mAbs as well as those published elsewhere has revealed convergence to the three major sites on the surface of GP including the glycan cap, mucin-like domain and the base of GP where the GP1 and GP2 subunits interact^29^. Unexpectedly, all the neutralizing mAbs target to the overlapping epitopes in the base of GP while those that do not neutralize or do not neutralize in the absence of complement bind to the glycan cap and mucin-like domains^29^,^30^,^31^,^32^,^33^,^34^,^35^,^36^. Most recent studies have identified several novel and cross-reactive mAbs with potent neutralizing activity and conferred protection in mouse, guinea pig or monkey models^33^,^37^,^38^,^39^,^40^,^41^. It needs to be noted, however, that neutralizing activities of mAb is not necessarily the predictor of protection *in vivo*^26^,^33^,^34^ and other mechanisms such as preventing budding of nascent viral particles from infected cells and Fc-mediated functions have to be taken into account^42^.

Here, we set out to raise neutralizing mAb targeting the GP of EBOV identified in 2014. Through sequential immunization of Chinese rhesus macaques and antigen-specific single B cell sorting, we have successfully identified three mAbs (Q206, Q314 and Q411) with potent neutralizing activities against pseudo and live EBOV. Epitope mapping through competitive binding, single-particle EM reconstructions and site-directed mutagenesis have revealed that Q206 and Q411 bind to novel and overlapping epitopes spanning the glycan cap and the head subdomain, approaching to the RBD of GP1 subunit. Q314, on the other hand, binds to an epitope that is partially overlapping with that of c13C6 in the glycan cap but is orientated towards the upper surface of the chalice. In distinct contrast to c13C6 and other mAbs binding to the similar or adjacent epitopes, the newly isolated mAbs are completely or partially independent of complement in live EBOV neutralization. In particular, all these novel mAbs are able to achieve close to 100% inhibition of live EBOV in the presence of complement while c13C6 plateaued just above 50% inhibition. Furthermore, treatment with these mAbs individualy provided partial but significant protection against disease progression in a mouse model of EBOV infection. Collectively, these results suggest that Q206 and Q411 recognize novel epitopes in the GP1 subunit with potent neutralizing activity and could serve as promising candidates for prophylactic and therapeutic interventions against Ebola virus infection.

## Results

### Generation and isolation of mAb from GPdM immunized macaques

We used recombinant GPdM with the deletion of mucin-like domain (MLD) and transmembrane domains to immunize two rhesus macaques (Rh091730 and Rh100888) largely due to the earlier studies indicating the bulky MLD is highly variable and able to shield the critical receptor binding domain (RBD) of Ebola virus glycoprotein^13^,^14^,^29^,^43^,^44^. To enhance and promote the maturation of the antibody response, eight consecutive immunizations were performed through intramuscular route at 4-week intervals before the blood samples were collected and evaluated for antibody binding and neutralization activities. Both animals mounted a strong antibody binding activity measured by ELISA as well as neutralizing activity using pseudovirus bearing the glycoprotein of EBOV Mayinga strain (supplementary Fig. 1). In particular, Rh100888 appeared to have 3-fold higher neutralizing activities than that of Rh091730 (527 vs. 156 in ID_50_ values) (supplementary Fig. 1b). We have therefore chosen the peripheral blood mononuclear cells (PBMCs) from Rh100888 to isolate GPdM-specific single memory B cells (CD3^−^, CD16^−^, CD235a^−^, CD19^+^, CD27^+^, CD38^−^, and IgG^+^) by FACS (supplementary Fig. 1c) and to amplify the variable regions of immunoglobulin heavy and light chain genes (VH and VL) by an optimized nested polymerase chain reaction (PCR). A total of 27 paired VH and VL sequences from the sorted single B cells were obtained and fused with the constant region fragment of human IgG1 to generate bivalent full-length chimeric mAbs. Once confirmed by sequencing, the full-length heavy and light chain plasmids were co-transfected into 293T cells for the production, purification and characterization of mAbs.

### Binding and neutralizing activity against pseudo and live Ebola virus infection

**Figure 1:**
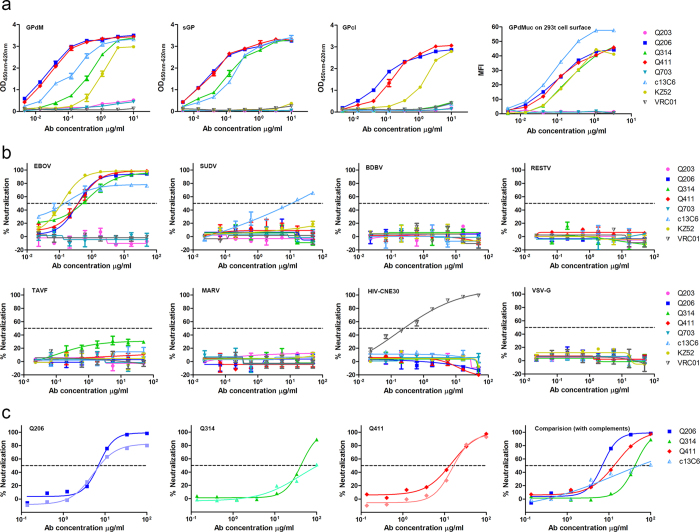
Binding (**a**) and neutralizing activities of isolated mAbs against pseudo (**b**) and live (**c**) Ebolaviruses. Pseudoviruses bearing the envelope glycoprotein from the five species of Ebolaviruses (EBOV, SUDV, BDBV, RESTV, TAFV) are presented as well as the controls from Marburg virus, human immunodeficiency virus (HIV) and vesicular stomatitis virus (VSV). Dashed line indicates 50% inhibition. Controls antibodies used here include VRC01, a human neutralizing mAb against HIV-1, c13C6 and KZ52, previously isolated neutralizing mAbs against EBOV. In live EBOV experiment (**c**), neutralizing activity was evaluated in the presence (dark) and absence (light) of complement in comparison with c13C6, one of the three mAbs in the ZMapp cocktail and its neutralizing activity is complement-dependent *in vitro*. Data presented are average values from at least two independent experiments and the error bars indicate for the standard error of the mean (SEM).

We first analyzed the binding activity of isolated mAbs to four different forms of EBOV GP including the recombinant GPdM, sGP, and GPcl as well as GP with deletion of MLD (GPdMuc) expressed on the surface of 293T cells. Among the total of 27 mAbs successfully isolated and produced, 5 (Q203, Q206, Q314, Q411 and Q703) had detectable binding to GPdM measured by ELISA. Q206, Q314 and Q411 demonstrated strong binding affinity comparable to that of the control mAbs c13C6 and KZ52 while Q203 or Q703 was merely above the detection threshold (Fig. 1a). Similar differences were also found in the binding to GPdMuc expressed on the surface of 293T cells, suggesting that binding of these mAbs is independent of MLD. Like c13C6, Q206, Q314 and Q411 bound strongly to sGP indicating their epitopes are located in the N-terminus of GP1 subunit where GPdM and sGP share amino acid residues between 31–295^29^. Interestingly, Q206 and Q411 maintained strong binding to the glycan cap missing GPcl while Q314 and c13C6 failed, suggesting that Q206 and Q411 recognized epitopes distinct from that by Q314 and c13C6. Genetically, each of the 5 mAbs had unique VH and VL sequences although Q206 and Q411 shared homologous sequence throughout except for the CDR3 sequence in VH (Table 1). The gene families of the 5 mAbs were quite divergent for both VH (1 V_H_1, 1 V_H_2, 2 V_H_3, and 1 V_H_4) and VL (3 V_λ_1, and 2 V_λ_2) with variable degree of divergence (90.1–95.3%) from the rhesus macaque germline sequences (Table 1). The degree of humanness of these recombinant full-length mAbs ranged from 97.3–99.7% by analyzing the amino acid sequence compared with human immunoglobulin gene using germline index^45^.

Neutralizing activity of Q203, Q206, Q314, Q411 and Q703 was then tested on the susceptible Vero-E6 cells against Ebola pseudovirus bearing the surface glycoprotein of EBOV, SUDV, BDBV, RESTV, or TAFV (Fig. 1b and Table 2). Consistent with binding activity, Q206, Q314 and Q411 demonstrated potent neutralization against EBOV (Mayinga strain) with IC_50_ values ranging from 0.36 to 0.78 μg/ml while Q203 and Q703 showed negligible effect (Fig. 1b and Table 2). Like KZ52, Q206, Q314 and Q411 were able to reach IC_90_ but c13C6 plateaued prematurely around 70% inhibition despite increases in concentration (Fig. 1b), consistent with earlier report^46^. Furthermore, differences in slope and AUC were also found among and between isolated and control mAbs, suggesting different mAbs demonstrated different potency and may exert their neutralizing activity through different mechanisms (Table 2)^47^. None of the isolated mAbs demonstrated any cross-neutralizing activity against the remaining species of Ebolaviruses (SUDV, BDBV, RESTV, or TAFV) or the pseudovirus bearing the envelope glycoprotein from MARV, HIV-1 or VSV. Only c13C6 displayed some level of cross-activity against SUDV strain as previously reported^32^. No cross-binding was found against GPdM from SUDV, BDBV and MARV (supplementary Fig. 2). VRC01, a human broadly neutralizing mAb against HIV-1^48^, demonstrated neutralizing activity against only HIV-CNE30 (Fig. 1b). Furthermore, Q206, Q314 and Q411 also showed potent neutralization against live EBOV virus (Fig. 1c and Table 2). In particular, Q206 and Q411 had similar IC_50_ concentrations in the presence or absence of complement suggesting their neutralizing activity is largely independent of complement. In contrast, Q314, like the mAb c13C6 control, required the presence of complement for optimal neutralization although significantly higher neutralization activity was found (Fig. 1c).

### Kinetics and competitive binding of isolated mAbs measured by SPR

**Figure 2:**
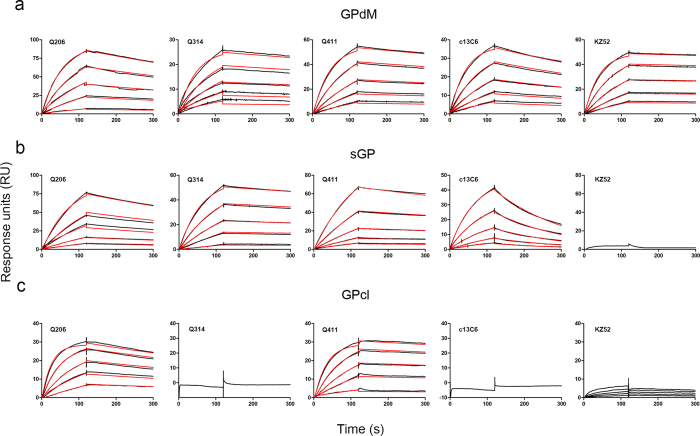
Binding kinetics of isolated mAbs with purified EBOV GPdM (**a**), sGP (**b**) and GPcl (**c**) measured by surface plasmon resonance (SPR). The purified soluble antigens GPdM, sGP and GPcl were covalently immobilized onto a CM5 sensor chip followed by injection of individual Fab of Q206, Q314, Q411 as well as control Fab c13C6 and KZ52 at five different concentrations. The black lines indicate the experimentally derived curves while the red lines represent fitted curves based on the experimental data.

The observed differences in neutralizing activity against pseudo and live Ebola virus could be due to their differences in binding affinity and epitopes recognized. To test this hypothesis, we measured the binding kinetics of Q206, Q314, and Q411 antigen-binding fragment (Fab) to the recombinant GPdM, sGP, or GPcl by surface plasmon resonance (SPR). As shown in Fig. 2, the differential binding to these recombinant proteins is consistent with those analyzed by ELISA (Fig. 1a). Furthermore, the estimated binding affinity to GPdM appeared to correlate with their neutralizing activity (Table 3). Q206 demonstrated the strongest binding affinity (*K*_d_ = 16.1 nM) while Q411 was intermediate (*K*_d_ = 24.0 nM) and Q314 was the weakest (*K*_d_ = 44.8 nM). Consistent with earlier report^49^, KZ52 demonstrated a higher binding affinity (*K*_d_ = 3.4 nM) than the 3 mAbs we isolated, hence also showing stronger neutralizing activity. However, c13C6 was an outlier perhaps due to its unique and unusual neutralization curve in that its inhibitory activity appeared early at the relatively lower concentration and then plateaued prematurely about 70% despite increases in concentration (Fig. 1b). In contrast, binding affinity to sGP didn’t seem to follow their trend in neutralization. Q314, the weakest neutralizing mAb isolated, showed the highest affinity (*K*_d_ = 6.3 nM) to sGP while Q206 demonstrated the intermediate (*K*_d_ = 7.1 nM) and Q411 was the weakest (*K*_d_ = 13.5 nM). Interestingly, removal of the glycan cap from GPdM by thermolysin (GPcl) dramatically increased the binding of Q206 (from *K*_d_ = 16.1 to 7.1 nM) while having no effect on Q411 (from *K*_d_ = 24.0 to 24.0 nM) (Table 3). These results highlight the binding affinity to membrane associated (GPdM and GPcl) but not secreted (sGP) form of GP may contribute to the differential neutralizing activity among the isolated mAbs. In particular, as EBOV entry requires cleavage of envelope glycoprotein in the endosome^13^,^15^, more favorable binding of Q206 to GPcl over GPdM suggests a possible enhanced inhibitory effect by this mAb during the process of viral entry.

**Figure 3:**
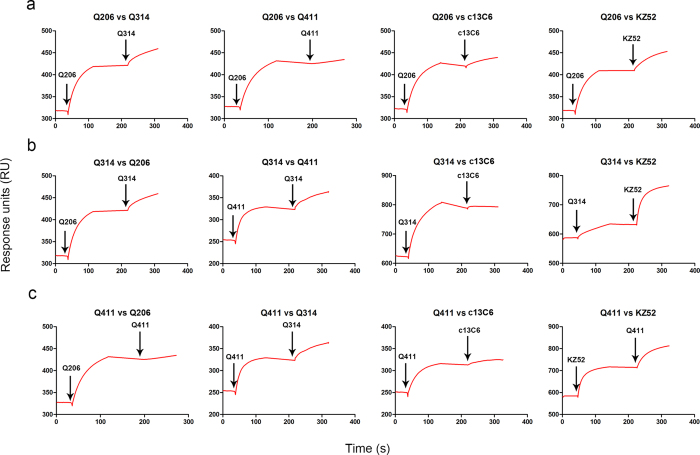
Epitope mapping through competitive binding measured by SPR. The sensorgrams show distinct binding patterns when pairs of testing Fabs were sequentially applied to the purified GPdM covalently immobilized onto a CM5 sensor chip. Arrows indicate the time points when Fabs were injected. Whether additional binding after injecting the second Fab is the key criteria for determining the two mAbs recognize the separate or closely situated epitopes.

We went further to determine the epitopes of the isolated mAbs on GPdM through pairwise competition using SPR. Specifically, pairs of the testing Fabs were sequentially applied to the purified GPdM covalently immobilized on a CM5 sensor chip to monitor for additional binding to determine whether the two mAbs recognized the separate or closely situated epitopes. As shown in Fig. 3a, Q206 did not compete with Q314 nor with KZ52, but strongly with Q411 and partially with c13C6. Q314, on the other hand, strongly competed with c13C6, but not with Q206, Q411 or KZ52 (Fig. 3b). Conversely, Q411 strongly competed with Q206, partially with c13C6 but not with Q314 or KZ52 (Fig. 3c). These results clearly indicate that Q206 and Q411 recognize an overlapping epitope and so did Q314 and c13C6. The two overlapping epitopes appeared to be closely located but grossly distinct from that recognized by KZ52.

### Single particle EM reconstructions of GPdM in complex with Q206, Q314 or Q411 Fab

**Figure 4:**
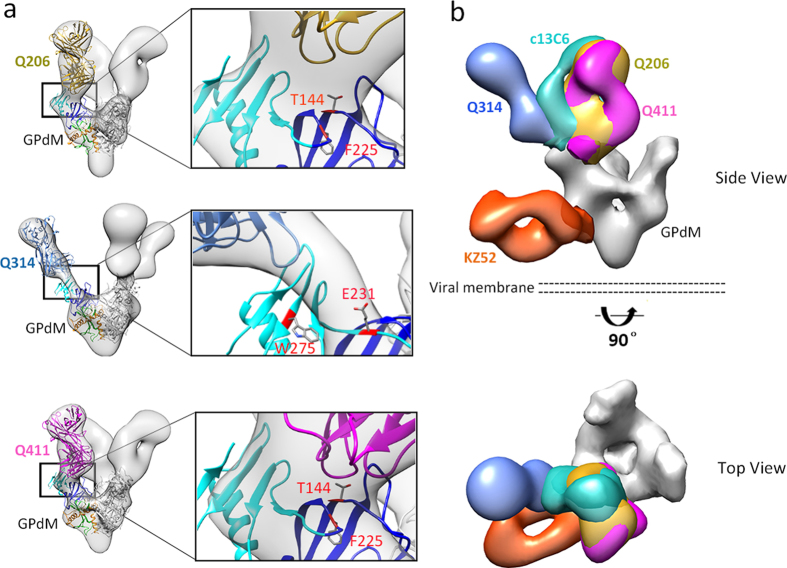
Single-particle EM reconstructions of Q206, Q314 and Q411 Fabs bound to EBOV GPdM. Hybrid models of negative-stain EM reconstructions fit with the EBOV GPdM crystal structures (PDB assessing number 3CSY) and a reference Marburg virus mAb MR78 Fab (PDB assessing number 3X2D). (**a**) Side view of individual Fab Q206 (mustard), Q314 (blue) and Q411 (magenta) bound to the EBOV GPdM. Overall as well as focused views at the binding interface between Fab and GPdM are shown in ribbon diagram. Four residues (T144, F225, E231 and W275) found to be critical for mAb binding and neutralization through mutagenesis study are highlighted and found to be located in the glycan cap (cyan) and the head regions (dark blue) within the EBOV GPdM. b) Side and top views of combined Fabs of Q206 (mustard), Q314 (blue), and Q411 (magenta) bound to the EBOV GPdM relative to the control Fabs c13C6 (cyan) and KZ52 (orange).

To further determine the epitopes in a more precise manner, negative stain single-particle EM reconstructions were performed with GPdM in complex with Fab of Q206, Q314 or Q411. Each Fab was added in excess to GPdM, purified by size-exclusion chromatography and then subjected to staining and EM analyses (Fig. 4, supplementary Fig. 3 and supplementary Table 1). The structures of Q206- and Q411-GPdM complexes showed that the antibodies bound to novel epitopes within the center of the GPdM chalice, perpendicularly to the expected plane of the membrane and making contacts with a region spanning the glycan cap (cyan) and the head (blue) subdomains of GP1 subunit (Fig. 4a and supplementary Fig. 3). Q314, on the other hand, bound in the vicinity of the glycan cap with an angle of approach much less steep than that of Q206 and Q411 (Fig. 4a and supplementary Fig. 3). To compare the binding sites of Q206, Q314 and Q411 to those of c13C6 and KZ52, we generated a hybrid map based on the structure information obtained here and previously published elsewhere^29^. As shown in Fig 4b, the binding sites for Q206 and Q411 are located next to that of c13C6 but closer to the inner chalice of the GP1 subunit. On the contrary, the Q314 binding sites are located to the opposite side of c13C6 in the glycan cap orientated towards the upper surface of the chalice (Fig. 4b). As shown previously, the control KZ52 bound the base of GPdM^14^,^29^. These results are consistent with those from the epitope mapping studies conducted above and provided a clear three dimensional understanding of the location and distribution of epitopes recognized by Q206, Q314 and Q411 on the GP1 subunit.

### Epitope mapping through site-directed mutagenesis

To confirm the epitope information obtained, we conducted site-directed mutagenesis on EBOV GPdMuc (Mayinga strain) based on the structural information as well as previously published studies shown to confer resistance to control mAbs c13C6 and KZ52^29^,^32^,^50^. A total of 23 single mutant clones were generated, one in the base, four in the head, thirteen in the glycan cap of GP1 subunit and five in the GP2 subunit (Table 4). Once confirmed by sequencing, the expressing vectors encoding the wild type and mutant clones were transfected into 293T cells and analyzed for the binding of Q206, Q314 and Q411 as well as c13C6 and KZ52 by FACS. Nine out of the 23 clones had profound impact on the binding activity of the mAbs. T144A and F225A mutations in the head, for instance, either completely abolished or significantly reduced the binding of Q206 and Q411. Mutant T144A also demonstrated significant reduction to Q314 binding. In addition, mutations in the glycan cap (E231A, T270A and W275A) had biased effect towards Q314 and c13C6. For example, both E231A and W275 mutants either abolished or significantly reduced the binding by Q314 and c13C6 while E231A also demonstrated negative effect on binding by Q206, Q411 and KZ52. T270A mutant, previously shown to affect c13C6 binding, resulted in the similar effect in our assay^32^. Lastly, consistent with previous reports^32^,^50^, mutations at Q508, C511, N550, and D552 in the GP2 had detrimental effect on KZ52 binding but minimal effect on the rest of the mAbs tested here.

**Figure 5:**
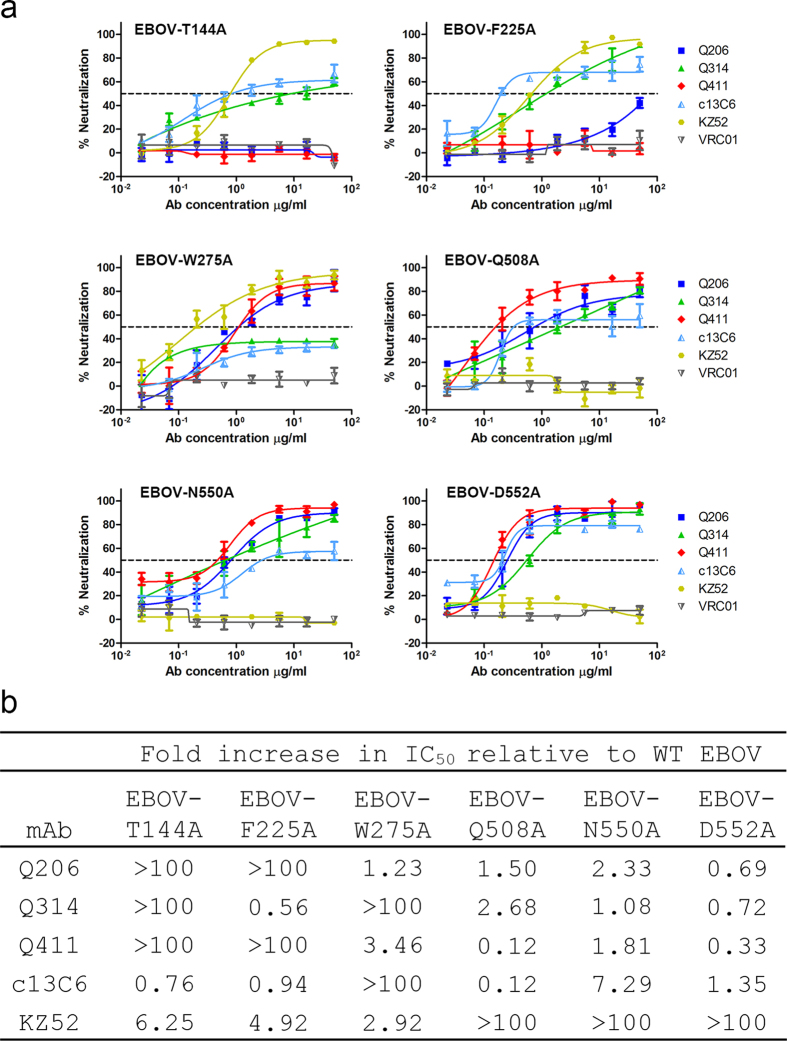
Critical residues on the EBOV envelope glycoprotein in conferring resistance to Q206, Q314 and Q411 neutralization. Comparison of neutralization sensitivity of mutant and wild-type pseudoviruses to Q206, Q314, and Q411 in graphic (**a**) and numerical (**b**) format. Dashed line indicates 50% inhibition. Controls antibodies used here include VRC01, a human neutralizing mAb against HIV-1, c13C6 and KZ52, previously isolated neutralizing mAbs against EBOV. Data presented are average values from at least two independent experiments and the error bars indicate for the standard error of the mean (SEM).

Next, we studied whether reduction in binding could confer resistance to mAb neutralization in the context of pseudovirus. To this end, the pseudoviruses bearing the representative mutations were generated and subjected to standard neutralization test (Fig. 5). Consistent with the binding analysis, T144A and F225A mutations in the head resulted in complete resistance to Q206 and Q411 neutralization with at least 100-fold increase in IC_50_ compared to the wild type. Similarly, W275A mutation in the glycan cap and Q508A, N550A and D552A in GP2 subunit also rendered pseudovirus completely resistant to Q314 and c13C6 as well as KZ52, respectively. It needs to be noted that the same set of mutations had some noticeable cross-effect on neutralization activity of other mAbs, but all limited within the 10-fold differences from the wild type and most likely due to the indirect consequences (Fig. 5b). But nevertheless, these results confirm that Q206 and Q411 bind to a novel epitope located between the glycan cap and the head subdomain approaching to RBD in the inner chalice of GP1. Q314, on the other hand, binds to an overlapping epitope with c13C6 in the glycan cap but more towards the upper surface of the chalice.

### Post attachment inhibition through partial interference with receptor NPC1 binding

**Figure 6:**
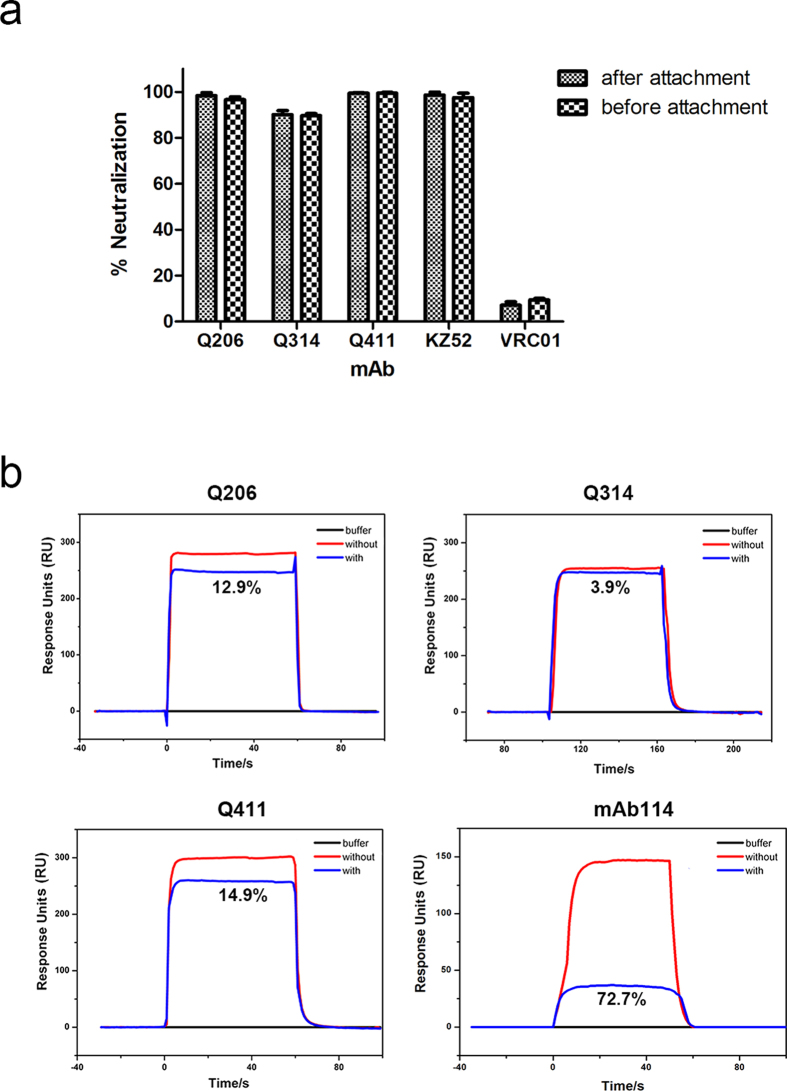
Post attachment inhibition through partial interference with receptor NPC1 binding. (**a**) The testing mAb were applied to pseudovirus and Vero-E6 cells before or after attachment and (**b**) Competitive binding of the testing mAbs with NPC1-C to immobilized GPcl on a chip. The blocking efficacy was determined as 12.9% for Q206, 3.9% for Q314, 14.9% for Q411, and 72.7% for control mAb 114, respectively.

To determine potential steps of inhibition, we applied the testing mAbs to pseudoviruses and target Vero-E6 cells in two separate time scales. One was to incubate with pseudoviruses first at 37 °C for 1 h before virus attachment to Vero-E6 cells. The other was allow virus attachment to Vero-E6 cells first at 4 °C for 1 h before adding the testing mAbs and elevating temperature to 37 °C to initiate the virus entry. We reasoned that if the testing mAb inhibited virus attachment to the cell surface, the reduction in luciferase activity would only be detectable in the former experiment. On the other hand, if the testing mAb inhibited the steps after virus attachment, the reduction in luciferase activity would be detectable in both cases. As shown in Fig. 6a, Q206, Q314 and Q411 were all able to reduce luciferase activity more than 90% in both cases, suggesting they inhibited steps after virus attachment. Consistent with earlier reports, the positive control KZ52 also inhibited post-attachment steps while the negative control VRC01 failed in both cases (Fig. 6a).

As the epitopes of Q206 and Q411 located between the glycan cap and the head subdomain approaching to the RBD, Q206 and Q411 may exert their neutralizing activity after attachment through interfering with GPcl binding to the cellular receptor NPC1. To test this hypothesis, we immobilized GPcl to a CM5 sensor chip followed by injection of Q206, Q314 or Q411 separately until reaching the binding steady-state. Domain C of NPC1 (NPC1-C) was then injected to compare the binding kinetics with or without the testing mAbs. As shown in Fig 6b, both Q206 and Q411 partially interfered with NPC1-C binding with efficacy about 12.9% and 14.9%, respectively. Q314, on the other hand, demonstrated negligible effect of 3.9%. The positive control mAb114, recently isolated and shown to block receptor NPC1 binding^41^, was about 72.7% effective. These results indicate that Q206, Q314 and Q411 inhibit virus entry at the post attachment steps. Q206 and Q411 appear to partially interfere with receptor NPC1 binding while Q314 functional step warrants further study.

### Protective efficacy in mouse model of EBOV infection

To determine the protective efficacy of isolated mAbs *in vivo*, we infected mice through intraperitoneal injection (IP) with a dose of 1000 × LD_50_ mouse-adapted EBOV and administered Q206, Q314 or Q411 at either 1 or 2 days post infection. All control animals, which received only PBS, succumbed to the virus with an average time of death of 7.4 ± 3.1 days. In contrast, mAb treatment significantly increased survival in infected mice (p-value = 0.028). The highest survival rates were 67% and 50% for the animal groups treated with Q206 48h (p-value = 0.0033) and Q411 at 24 h (p-value = 0.0169) (Table 5). Interestingly, the degree of weight change did not correlate with protective efficacy. In fact, the two groups with the highest survival also had the highest weight loss (supplementary Fig. 4).

### Discussion

The unprecedented EBOV outbreak in West Africa in 2014 has affected more people than all previously recorded outbreaks combined and highlighted the threat to global health^1^,^51^,^52^. While significant process has been made in therapeutics and vaccines against EBOV, no licensed products are currently available^18^,^19^,^22^,^23^,^24^,^25^. Combination of monoclonal antibodies represent one of the most specific and promising therapeutic modalities^22^,^23^,^24^,^25^ and one such cocktail ZMapp has demonstrated safety and efficacy in nonhuman primates as well as encouraging results in compassionate use in humans although the development for clinical application requires further studies^24^,^28^. These antibodies, together with most of the published ones, are directed to several vulnerable sites on the surface of GP including the glycan cap, mucin-like domain and the base of GP where the GP1 and GP2 interact^14^,^29^. All neutralizing mAbs, however, target to the overlapping epitopes in the base of GP while those do not neutralize or do not neutralize in the absence of complement bind to the glycan cap and mucin-like domains^14^,^29^,^31^,^32^,^36^. The most recent studies have isolated a large number of mAbs from convalescent individuals with neutralizing activities targeting to additional sites on the GP^38^,^39^,^40^.

This study aimed to identify neutralizing mAbs targeting additional sites on the GP of Ebola virus identified in 2014^1^. Through sequential immunization of Chinese rhesus macaques with recombinant GPdM and antigen-specific single B cell sorting, we have successfully isolated and characterized three mAbs (Q206, Q314 and Q411) with potent neutralizing activities against pseudo and live EBOV. Epitope mapping through differential binding, single-particle EM reconstructions and site-directed mutagenesis have revealed that Q206 and Q411 bind to novel and overlapping epitopes spanning the glycan cap and the head subdomain approaching to the RBD in the inner chalice of GP1. Q314, on the other hand, binds to an overlapping epitope with c13C6 in the glycan cap but orientated towards the upper surface of the chalice. In distinct contrast to c13C6 and other mAbs binding to the similar or adjacent epitopes, the newly isolated mAbs are completely or partially independent of complement in live EBOV neutralization. In particular, all these novel mAbs are able to achieve close to 100% inhibition of live EBOV in the presence of complement while c13C6 plateaued just above 50% inhibition. Furthermore, treatment with these mAbs provided partial but significant protection against disease progression in a mouse model of EBOV infection. Collectively, these results suggest that Q206 and Q411 recognize novel epitopes in the GP1 with potent neutralizing activity and could serve as promising candidates for prophylactic and therapeutic interventions against Ebola virus infection.

We believe that the neutralizing mAbs identified here represent a new arsenal against EBOV infection. The most potent and protective mAbs, Q206 and Q411, recognize overlapping epitopes within the core of GP1, a region that encompasses the RBD and the base of GP1^14^,^29^,^32^. While previous studies suggested that this region may be concealed before proteolysis by endosomal cathepsins and receptor binding^15^,^29^,^53^, the binding as well as neutralizing profile of Q206 and Q411 indicated this region contains opening spots for antibody binding and neutralization at the early steps during viral entry. While the exact underlying mechanism requires further investigation, it appeared that Q206 and Q411 exert their neutralization activity through interfering with GP binding to its receptor NPC-1 which may lead to affecting the subsequent fusion between viral and cellular membrane. This hypothesis is further supported by the mutagenesis study where mutant virus with T144, a residue located at the edge of RBD critical for NPC-1 binding^54^, became resistant to Q206 and Q411 (Fig. 5 and Table 4). For Q314, however, the mechanism of action is far less clear. Despite of its overlapping epitope with c13C6, Q314 protective activity in the mouse model is much less effective^31^, perhaps due to the differences in exact epitope, the affinity, the approaching angle or the Fc-mediated functions of the antibody to the GP. Of note, it is important to recognize that neutralizing activity does not always translate into protective activity in mouse model and mouse model can’t always predict the monkey model. The ultimate test will have to be conducted in human before any meaningful protective activity could be evaluated and determined. In any case, it would be interesting to study whether the combination of our newly isolated mAbs could have any synergistic effect to provide stronger and broader neutralizing and protective activities against wild-type and mutant EBOV^14^,^22^,^23^,^24^,^25^,^33^,^34^,^35^,^36^,^49^. Although the current dataset for EBOV shows a limited degree of sequence variation, this is by no means to suggest the virus will remain unchanged in the long run given their wide spread in several animal species, continuing zoonotic introductions into and rapid transmission within human population^7^,^8^,^12^,^50^,^55^,^56^,^57^. With the potent neutralizing mAbs identified here and their novel epitope specificities, we are at more advantageous position to generate and optimize next-generation of therapeutic antibody cocktail against EBOV infection.

## Methods

### Ethics statement

Rhesus macaque experiment was carried out in strict compliance with Chinese government rules and regulations for animal health and welfare. The experimental protocol for immunization and blood collection at Guangzhou Institute of Biomedicine and Health, Chinese Academy of Sciences were approved by the Institutional Animal Care and Use Committee. Mice experiments were performed at the National Microbiology Laboratory in Winnipeg, Canada. All animal experiments have been approved by the Animal Care Committee at the Canadian Science Center for Human and Animal Health in accordance with the guidelines outlined by the Canadian Council on Animal Care.

### Immunization of rhesus macaques

Two 5–6 year old Chinese rhesus macaques (Macaca mulatta) (Rh091730 and Rh100888) were immunized intramuscularly about every 4 weeks with 100 or 200 μg of recombinant EBOV GP devoid of mucin-like and transmembrane domains (GPdM) derived from the Guinean patient C7 (Genbank accession number KJ660347) identified in the 2014 outbreak^1^. The first two doses were formulated with alum adjuvant (Imject Alum, Thermo Scientific) while the subsequent doses were recombinant GPdM only. Plasma and peripheral blood mononuclear cells (PBMCs) were collected after the 8^th^ immunization, evaluated for binding and neutralization activity and used for sorting of single B cells and amplifying immunoglobulin heavy and light chain genes (VH and VL) by nested polymerase chain reaction (PCR). The animals were housed in the Animal Experimental Center of the Guangzhou Institute of Biomedicine and Health, Chinese Academy of Science.

### Production and purification of Ebola virus glycoprotein

All forms of EBOV GP (GPdM, sGP and GPcl) were derived from the Guinean patient C7 (Genbank accession number KJ660347) identified in 2014 outbreak and shared approximately 97% homology with Zaire strain from the Democratic Republic of Congo and Gabon^1^. Specifically, GPdM with the deletion of mucin-like (MLD) and transmembrane domains (residues 1–32, 312–463 and 633–676) was cloned into the pFastBac1 vector (Invitrogen) as previously described^14^. For protein secretion and purification, a gp67 signal peptide and a His_6_-tag were added at the N-terminus and C-terminus of the protein, respectively. Transfection and virus amplification were performed according to manufacture instructions (Invitrogen)^58^. Soluble GPdM was produced in Hi5 cells (Invitrogen), harvested from the culture supernatants by metal affinity chromatography using a HisTrap^®^ HP 5-ml column (GE Healthcare), and purified by gel filtration on a HiLoad 16/60 Superdex^®^200 PG column (GE Healthcare). GPcl with glycan cap removed was generated by treating the GPdM (2 mg/ml) with thermolysin (Sigma, 0.5 mg/ml) in HEPES-MES buffer (20 mM HEPES, 20 mM morpholinepropanesulfonic acid, 130 mM NaCl, pH 7.5) at 37 °C for 1 h. The cleavage was stopped by the addition of EDTA to final concentration of 10 mM.

The gene encoding the sGP (residues 33–324) was cloned into the baculovirus transfer vector pFastBac1 (Invitrogen) in-frame with an N-terminal gp67 signal peptide for secretion and a His6-tag at the C terminus for purification. Transfection and virus amplification were performed according to manufacture instructions (Invitrogen). Recombinant proteins were produced by infecting suspension cultures of Hi5 cells (Invitrogen) for 2 days, recovered from cell supernatants by metal affinity chromatography using a HisTrap HP 5-ml column (GE Healthcare), and purified by gel filtration chromatography using a Superdex-200^®^ 16/60 GL column (GE Healthcare) with a running buffer of 20 mM Tris-HCl, pH 8.0, 150 mM NaCl.

### PCR amplification of antibody heavy and light genes from sorted single B cells

Peripheral blood mononuclear cells (PBMCs) of immunized rhesus macaques were isolated from the whole blood by standard Ficoll-Hypaque (GE Healthcare) separations, and subsequently stained with various cell surface markers to identify, enrich and sort for single antigen-specific memory B cells (CD3^−^, CD16^−^, CD235a^−^, CD19^+^, CD27^+^, CD38^−^, IgG^+^ and GPdM-positive) using FACS Aria II flow cytometer (BD Biosciences, USA). The sorted single B cells in the 96 well plate were then reverse transcribed and variable regions of immunoglobulin heavy and light chain genes (VH and VL) were amplified by nested PCR using an optimized version of a previously described protocol^59^. In brief, the reverse transcription reaction was performed with mixture of SuperScript III reverse transcriptase, RNase Out, dNTPs (Invitrogen), primers specific for IgG, IgM, IgA, IgD, IgE, Igκ, and Igλ, and incubated at 55 °C for 60 min. The cDNA was then amplified by PCR with appropriate primer pairs in a 50 μl reaction at 95 °C for 5 min followed by 35 cycles of 94 °C for 30 s, 67 °C for 45 s, 72 °C for 90 s with final extension of 72 °C for 7 min. The second PCR was performed in exactly the same conditions except for the primer pairs located within the internal region compared to those used in the first PCR. PCR products were analyzed on a 1.0 % agarose gel, purified and sequenced for confirmation.

### Expression and purification of recombinant antibody

The PCR-amplified and sequence-confirmed the variable regions of heavy and light chain genes were separately cloned into backbone of antibody expression vectors containing the constant regions of human IgG1^60^. Whole recombinant human IgG1 antibodies were expressed in 293T cells (ATCC) by transient transfection and purified by affinity chromatography using Protein A agarose. The concentration was determined by BCA Protein Assay Kit (Thermo Scientific). The expression clones encoding the control antibody KZ52, c13C6, mAb114 were synthesized and confirmed by sequencing before production.

### Analysis of antibody binding through ELISA and cell surface expressed glycoprotein

For enzyme-linked immunosorbent assay (ELISA), purified EBOV GP (GPdM, sGP, and GPcl) were immobilized at 100 ng/well in phosphate-buffer saline (PBS) on 96 well plates at 4 °C overnight. After blocking with 1% BSA, serial dilutions of purified antibodies were added and the bound antibodies were detected by anti-human immunoglobulin G (IgG)-horseradish peroxidase (Promega) and TMB (3, 3′, 5′, 5′- tetramethylbenzidine) substrate (CWBio). Absorbance was measured at 450 nm with background blank measured at 620 nm. For cell surface binding analysis, the expressing vector pcDNA3.1 encoding the gene of EBOV GPdMuc with the deletion of MLD (Mayinga strain) was transfected into 293T cells. Approximately forty hours later, the transfected cells were harvested, trypsinized, fixed with 4% paraformaldehyde, stained with a serial dilution of testing and control mAbs, detected by the anti-human IgG-Alexa Fluor 488 secondary antibody (Santa Cruz Biotechnology) using a FACS Calibur flow cytometer (BD Biosciences).

To measure the antibody cross-reactive against additional Ebolavirus species, Costar half-area high binding assay plates (Corning #3690) were coated at 4 °C overnight with 30 ng of EBOV GPdTM, EBOV sGP, SUDV GPdTM, BDBV GPdTM and MARV GPdTM (IBT BioServices, USA) diluted in 30 μl of PBS, respectively. Plates were blocked for 1 h with 5% skim milk/PBS at 37 °C. Testing mAbs were serially diluted in 2% skim milk/PBS (30 μl/well) and incubated for 2 h at 37 °C. After wash 6 times with PBS/0.1% Tween-20, 30 μl/well of a secondary antibody (horseradish peroxidase (HRP)–conjugated goat anti-human IgG) (KPL #474–1006, USA) was added at 0.5 μg/ml in 2% skim milk/PBS and incubated for 1 h at 37 °C. Following wash with PBS/0.1% Tween20, 50 μl/well of the HRP substrate TMB (ThermoFisher #00-2023) was added and incubated at room temperature for 30 min in the dark. Plates were read with the VersaMax Microplate Reader (Molecular Devices) using the SoftMax Pro 4 software, and results were reported as the optical density measured at 650 nm. At every step the plates were sealed with sealing film (Excel Scientific #100SEALPLT).

### Ebola pseudo and live virus neutralization assay

A total of five different species of Ebola pseudovirus (EBOV, SUDV, BDBV, RESTV, and TAFV) were generated by co-transfection of human immunodeficiency virus backbone expressing firefly luciferase (pNL43R-E-luciferase) and expression vectors pcDNA3.1 (Invitrogen) encoding the full-length glycoprotein of each Ebola specie into the 293T cells (ATCC). Viral supernatants were collected 48 h later, and viral titers were measured in luciferase activity in relative light units (Bright-Glo Luciferase Assay vector System, Promega Biosciences). The mutant EBOV glycoprotein was generated by the site-directed mutagenesis on the expression vector encoding the wild type EBOV glycoprotein (Mayinga strain) following the manufacture instructions (Agilent). All mutant clones were confirmed by sequencing. Control envelope glycoprotein from Marburg virus (MARV), human immunodeficiency virus (HIV-CNE30) and vesicular stomatitis virus (VSV) and corresponding pseudoviruses were made in the same manner. Controls mAbs included VRC01, a human neutralizing mAb against HIV-1^48^, c13C6, KZ52, mAb 114 previously isolated neutralizing mAbs against EBOV^31^,^39^,^49^. Neutralization assays were performed by incubating pseudovirus with 8 serial 3-fold dilutions of purified testing mAbs at 37 °C for 1 h. Vero-E6 cells (about 1.5 × 10^4^ per well) (ATCC) were infected in triplicate with the virus-antibody mixture. Neutralizing activities of the testing mAbs was determined by the luciferase activity 48 hours after infection and presented as IC_50_, IC_90_, slope calculated by the dose-response inhibition function in GraphPad Prism 5 (GraphPad Software Inc.) and the area under the curve (AUC) determined by MATLAB (MathWorks).

The fluorescent neutralization assay was performed in 96-well tissue culture plates (Corning) as previously described^24^,^50^. The virus, EBOV/May-eGFP (passage 4), was incubated with serial dilution of testing mAbs, ranging from 0.137 to 300 μg/ml, for 1 h at 37 °C in plain DMEM with or without complement (Sigma). Vero-E6 cells (ATCC) at 90–100% confluence were infected in triplicate with the virus-antibody mixture at an MOI of 0.1 TCID_50_ per cell. Infection was carried out for 1 h at 37 °C, 5% CO_2_, and the inoculum was then removed and replaced with DMEM/2% FBS. Plates were incubated for 72 h before fluorescent intensities of GFP were measured using a Synergy HT microplate reader (Biotek). The median EBOV neutralization concentration was calculated based on a four-parameter curve fitted to the EBOV/May-eGFP fluorescence curves using GraphPad Prism 5.

### Antibody binding kinetics, epitope mapping and competition with receptor NPC1 measured by SPR

The binding kinetics and affinity of mAbs to the three purified EBOV antigens GPdM, sGP, and GPcl were analyzed by surface plasmon resonance (SPR) (Biacore T200, GE Healthcare). The purified soluble antigens were covalently immobilized to a CM5 sensor chip via amine group in 10 mM sodium acetate buffer (pH 5.5) for a final RU around 350. All mAbs were cleaved to antigen-binding fragments (Fabs) by incubating with protease Lys-C (Sigma) at a ratio IgG:LysC = 4000:1 (w/w) in 10 mM EDTA, 100 mM Tris/Cl^−^, pH 8.5 at 37 °C for about 12 hours. SPR experiments were run at a flow rate of 30 ml/min in PBS buffer. The sensograms were fit in a 1:1 binding model with BIA Evaluation software (GE Healthcare). For epitope mapping, two different Fabs were injected sequentially to monitor for additional binding activity to determine whether the two mAbs recognized the separate or closely situated epitopes. For competition experiment with the domain C of NPC1 (NPC1-C), GPcl was immobilized to a CM5 sensor chip via amine group for a final RU around 2000. 1 μM of antibodies were injected onto the chip for 300 seconds until reaching a binding steady-state and then 100 μM of NPC1-C was injected for 60 seconds. The blocking efficacy was determined by the comparison of response units with and without antibodies pre-incubation. NPC1-C was produced and purified as previously reported^54^.

### Electron microscopy analysis of GPdM and Fab interaction

Purified EBOV GPdM was incubated with Fab of the testing mAbs in a molar ratio of 1:4 at 4 °C for about 12 h and the formed complex was purified with size exclusion chromatography. Four microliters of each GPdM-Fab complex at a concentration of ~0.02 mg/ml was applied onto a glow discharged continuous carbon grid (Beijing Xinxing Braim Technology Co., Ltd.). Once enough materials were adsorbed, the grid was treated with filter paper to remove the excess sample, immediately washed twice and incubated with ~4 μl of 1% uranyl acetate (UA) solution for additional 30 s. The grid was then further treated with filter paper to remove the UA, air-dried at room temperature, and examined under an FEI Tecnai F20 electron microscope equipped with an FEG filament and operated at 200-kV acceleration voltage, using a nominal magnification of 50,000× at a pixel size of 0.168 nm.

### Electron microscopy image processing

Images were recorded using a Gatan 895 4 k × 4 k CCD camera with an exposure dose of 20–30 e^−^/Å^2^. The defocus range used was -1 μm to -3.5 μm. EMAN2 software package^61^ was used to pick the particles and generate the initial models. Several rounds of reference-free 2D classification and 3D classification were performed to select good particles using the program Relion1.4^62^. The selected particles were then used for the 3D auto-refinement and reconstruction with a C3 symmetry imposed. The resolution of the final map was calculated using the golden standard Fourier shell correlation value at 0.143. EM maps and related materials have been deposited to the EM Data Bank under accession codes EMD-8158, EMD-8159 and EMD-8160. To better interpret the EM maps, crystal structures of the EBOV GPdM (PDB accessing number 3CSY) and a Marburg virus mAb MR78 Fab (PDB accessing number 3X2D) were fitted into the EM density maps with the programs Situs^63^. The EBOV GPdTM, Fabs c13C6, KZ52 EM map (EMDB accessing number 6153) was fitted with our EM density maps using Chimera^64^.

### Protective efficacy of mAbs in mice

BALB/c mice, 4–6 week old, female and weighing between 15–19 g, were randomly assigned into groups of 5–6 mice. All mice were challenged through intraperitoneal injection (IP) with a dose of 1000 × LD_50_ mouse-adapted EBOV in 200 μL DMEM. The mAb were given IP once at 1 or 2 days post infection with 100 μg of each individual mAb per mouse. The control group was given the same volume of PBS. All animals were monitored for signs of disease, survival and weight change for 16 days, and survival was monitored for 12 additional days.

### Statistical analysis

In Ebola pseudovirus neutralization experiments, half-maximal and ninety percent inhibitory concentrations (IC_50_ and IC_90_) as well as inhibitory slope for each mAb were calculated using the dose-response inhibition model in GraphPad Prism (GraphPad Software Inc.). The area under the curve (AUC) for each mAb was determined by MATLAB (MathWorks). In EBOV live virus neutralization experiments, the median neutralization concentration for each mAbs was calculated based on a four-parameter curve fitted to the EBOV/May-eGFP fluorescence curves using GraphPad Prism 5. In mice challenge and protection experiments, differences in survival were calculated for each group compared to those receiving PBS using a log-rank (Mantel-Cox) test in GraphPad Prism 5 with * means P < 0.05 and ** means P < 0.01, respectively.

## Additional Information

**How to cite this article**: Zhang, Q. *et al*. Potent neutralizing monoclonal antibodies against Ebola virus infection. *Sci. Rep.* **6**, 25856; doi: 10.1038/srep25856 (2016).

## Supplementary Material

Supplementary Information

## Figures and Tables

**Table 1:** Genetic analysis of the heavy and light chain variable regions of isolated mAbs.

	mAb	V	Variable region identity(%)*	Full length identity (%)†	CDR1	CDR2	CDR3
VH	Q203	V_H_2.12	95.3	98.7	GFSLSTSGMG	IYWDDDKR	VRWAPPSAMLGDAFDFWG
	Q206	V_H_3.9	93.0	99.7	GFTFSTFW	IKTKPDGGTA	TTRQRAHDYWG
	Q314	V_H_4.26	90.5	98.8	GASISSYW	INGNSGIT	ARRSQGSLDWLLDHGLDSWG
	Q411	V_H_3.9	94.3	99.2	GFTFSSVW	IKSKADGGTP	TTRITTFALILNRFDVWG
	Q703	V_H_1.53	92.2	98.3	GYTFTDYY	VDPEDGDV	TAGGSGNSNWFHVWG
VL	Q203	V_λ_2.13	94.3	97.3	SSDIGGYNY	EVN	SSYVDSHNLLFG
	Q206	V_λ_1.30	94.6	98.6	SSNIGAGYS	END	QSYDSRMSDNAVFG
	Q314	V_λ_1.27	90.1	97.7	RSNIGADN	HSN	AAWDDSLSSMLFG
	Q411	V_λ_1.30	94.6	97.6	SSNIGTGYG	ENN	QSYDSSLSDHYVFG
	Q703	V_λ_2.13	95.3	98.6	SSDIGGYNY	EVN	SSYVDSHSLLFG

^*^Variable region identity to Rhesus germline (%).

^†^Full length identity to Human germline (%).

**Table 2:** Neutralizing activity of isolated mAbs against pseudo and live Ebola virus.

EBOV pseudovirus*					EBOV live virus†			
mAb	IC_50_ (μg/ml)	IC_90_ **(μg/ml)**	Slope (m)	AUC (μg/ml)	IC_50_ (μg/ml)	IC_90_ (μg/ml)	Slope (m)	AUC (μg/ml)
Q203	>50	>50	n.a.	n.a.	n.a.	n.a.	n.a.	n.a.
Q206	0.36	1.49	1.54	234.01	7.08/5.16	17.66/n.a.	2.41/1.62	125.03/84.12
Q314	0.78	6.77	1.02	239.29	42.96/38.90	91.20/n.a.	2.24/0.91	45.09/29.17
Q411	0.43	2.23	1.33	247.34	14.92/15.24	61.66/61.65	1.40/1.93	105.37/62.40
Q703	>50	>50	n.a.	n.a.	n.a.	n.a.	n.a.	n.a.
c13C6	0.17	n.a.	1.04	247.29	54.95/n.a.	n.a./n.a.	0.41/n.a.	54.35/n.a.
KZ52	0.12	0.58	1.41	291.42	n.d.	n.d.	n.d.	n.d.

^*^Neutralization activity is presented by four different parameters: IC_50_, IC_90_, Slope and Area under the curve (AUC).

^†^Neutralization activity was evaluated with and without complement and expressed numerically before and after the forward slash, respectively.

n.a.: not applicable. n.d.: not determined.

**Table 3:** Binding kinetics of isolated mAbs with purified GPdM, sGP and GPcl of EBOV.

mAb	GPdM*			sGP			GPcl		
	K_on_ (M^−1^s^−1^)	K_off_ (s^−1^)	K_d_ (M)	K_on_ (M^−1^s^−1^)	K_off_ (s^−1^)	K_d_ (M)	K_on_ (M^−1^s^−1^)	K_off_ (s^−1^)	K_d_ (M)
Q206	7.30E + 04	1.17E − 03	1.61E − 08	1.92E + 05	1.36E − 03	7.08E − 09	1.55E + 05	1.12E − 03	7.21E − 09
Q314	1.10E + 04	4.94E − 04	4.48E − 08	6.80E + 04	4.31E − 04	6.33E − 09	–	–	–
Q411	2.21E + 04	5.32E − 04	2.40E − 08	7.71E + 04	1.04E − 03	1.35E − 08	1.52E + 04	3.64E − 04	2.40E − 08
c13C6	4.60E + 04	1.39E − 03	3.02E − 08	7.83E + 04	5.12E − 03	6.54E − 08	–	–	–
KZ52	5.26E + 04	1.77E − 04	3.37E − 09	–†	–	–	–	–	–

^*^K_on_ (association rate, in M^−1^s^−1^), *K*_off_ (dissociation rate, in s^−1^), and *K*_d_ (dissociation constant, in M) are indicated for each paired interaction.

^†^Dash sign indicates the negative binding.

**Table 4:** Impact of mutant residues on mAb binding to surface expressed GPdMuc.

GPdMuc mutants	Location	Q206*	Q314	Q411	c13C6	KZ52
W104A	Base	++	++	++	++	++
D117A	Head	++	++	++	++	++
T144A	Head	−	+	−	++	++
P146A	Head	++	++	++	++	++
F225A	Head	+	++	+	++	++
N228A	Glycan cap	++	++	++	++	++
T227A	Glycan cap	++	++	++	++	++
E231A	Glycan cap	+	−	+	−	+
V236A	Glycan cap	++	++	++	++	++
L253A	Glycan cap	++	++	++	++	++
L254A	Glycan cap	++	++	++	++	++
T270A	Glycan cap	++	++	++	+	++
W275A	Glycan cap	++	−	++	+	++
K276A	Glycan cap	++	++	++	++	++
P279A	Glycan cap	++	++	++	++	++
T283A	Glycan cap	++	++	++	++	++
N296A	Glycan cap	++	++	++	++	++
L297A	Glycan cap	++	++	++	++	++
Q508R	GP2	++	++	++	++	−
C511A	GP2	++	++	++	++	−
N550A	GP2	++	++	++	++	−
D552A	GP2	++	++	++	++	−
G553A	GP2	++	++	++	++	++

^*^“+” indicates at least 50% reduction in fluorescent intensity compared to the wild type and “−” indicates complete abolish of antibody binding.

**Table 5:** Protective efficacy of mAbs against mouse-adapted EBOV in mice.

Group	Surviving/Total animals	Survival (%)	Mean time to death (days) ± SD
PBS	0/5	0	7.4 ± 3.1
Q206 24h	3/6	50.0	12.0 ± 4.4
Q206 48h	4/6	66.6	14.0 ± 3.6
Q314 24h	2/6	33.3	10.2 ± 4.6
Q314 48h	2/6	33.3	9.5 ± 5.3
Q411 24h	3/6	50.0	12.3 ± 4.1
Q411 48h	1/6	16.7	8.5 ± 3.7
