# Supplementary material for: Potent neutralizing monoclonal antibodies against Ebola virus infection
Source: Sci Rep. 2016 May 16;6:25856. doi: 10.1038/srep25856 (PMC4867612; doi:10.1038/srep25856)
Supplement: Supplementary Information [file srep25856-s1.pdf]

Potent neutralizing monoclonal antibodies against Ebola virus infection

Qi Zhang[1]*, Miao Gui[2]*, Xuefeng Niu[3]*, Shihua He[4,5], Ruoke Wang[1], Yupeng Feng[6], Andrea Kroeker [4,5] ,Yanan Zuo[1], Hua Wang[1], Ying Wang[1], Jiade Li[1], Chufang Li[3], Yi Shi[7], Xuanling Shi[1], George F. Gao[7], Ye Xiang[2], Xiangguo Qiu[4,5], Ling Chen[3,6], and Linqi Zhang[1]

[1] Comprehensive AIDS Research Center, and Collaborative Innovation Center for Diagnosis and Treatment of Infectious Diseases, School of Medicine, Tsinghua University, Beijing 100084, China.

[2] Beijing Advanced Innovation Center for Structure Biology, and Collaborative Innovation Center for Diagnosis and Treatment of Infectious Diseases, School of Medicine, Tsinghua University, Beijing 100084, China.

[3] State Key Laboratory of Respiratory Disease, First Affiliated Hospital of Guangzhou Medical University, Guangzhou 510230, China.

[4] Special Pathogens Program, National Microbiology Laboratory, Public Health Agency of Canada, Winnipeg, Manitoba, R3E 3R2 Canada

[5]Department of Medical Microbiology, University of Manitoba, Winnipeg, Manitoba, R3E 0J9 Canada

[6] Guangzhou Institute of Biomedicine and Health, Chinese Academy of Sciences, Guangzhou, 510530, China.

[7] CAS Key Laboratory of Pathogenic Microbiology and Immunology, Institute of Microbiology and Research Network of Immunity and Health, and Beijing Institutes of Life Science, Chinese Academy of Sciences, Beijing 100101, China.

*Co-first authors and Correspondence should be addressed to:

Linqi Zhang: zhanglinqi@tsinghua.edu.cn,

Ling Chen: chen_ling@gibh.ac.cn

Xiangguo Qiu: xiangguo.qiu@phac-aspc.gc.ca

Supplementary Fig. 1. Detection of binding (a) and neutralizing (b) antibody in immunized monkeys before sorting for GPdM-specific B cells (c). Antibody binding activity was measured against recombinant GPdM by ELISA and neutralizing activity was measured by pseudovirus bearing the glycoprotein of EBOV Mayinga strain. GPdM-specific memory B cells were identified through the combination of the following surface markers $CD3^{-}CD16^{-}CD235a^{-}CD19^{+}CD27^{+}CD38^{-}IgG^{+}$.

Supplementary Fig. 2. Cross-reactivity of mAbs against GPdM derived from variable Ebolavirus species. Antibody binding activity was measured against recombinant GPdM derived from EBOV, SUDV, BDBV and MARV by ELISA.

Supplementary Fig. 3. Negative-stain EM data. Representative negative-stain 2D class averages of the EBOV GPdM bound to Fab Q206 (a), Q314(c), or Q411(e). Golden standard Fourier shell correlation (FSC) curves used to determine the resolution of the final reconstructions. The final density maps are shown as side view along with the corresponding FSC curves. Bar=10nm.

Supplementary Fig. 4. Protective efficacy of mAbs in mice. Groups of 5-6 BALB/c mice were infected with 1000x LD50 of MA-EBOV via IP injection. At 1 or 2 dpi, mice received each monoclonal antibody in a single IP injection containing 100 µg mAb. **a)** The survival and **b)** percentage weight change are shown.

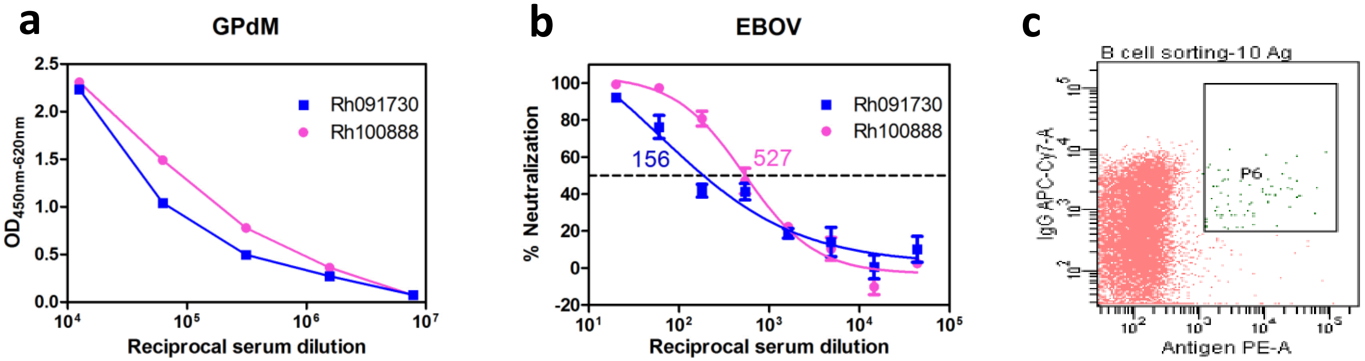

Supplementary Fig. 1

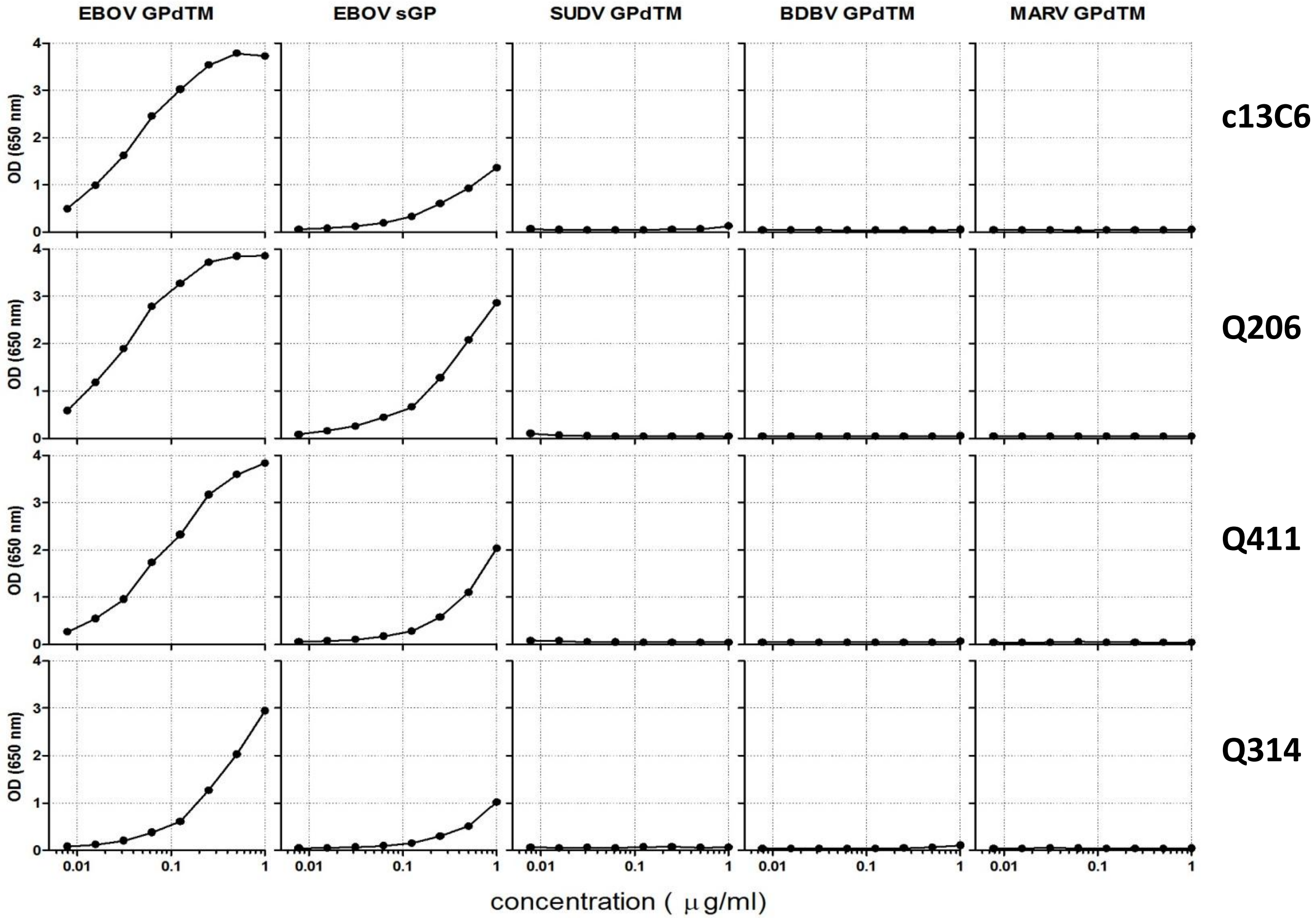

Supplementary Fig. 2

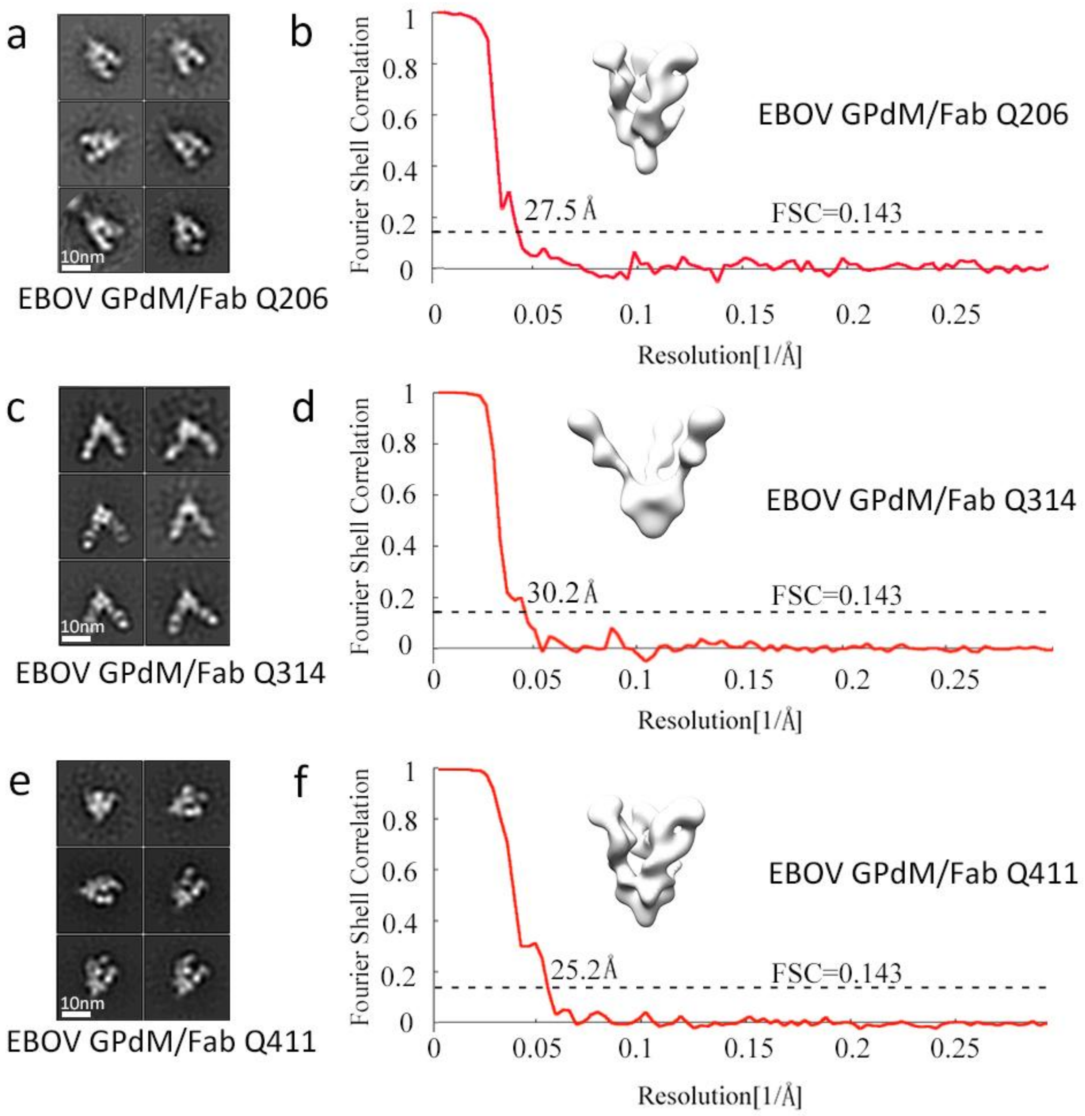

Supplementary Fig. 3

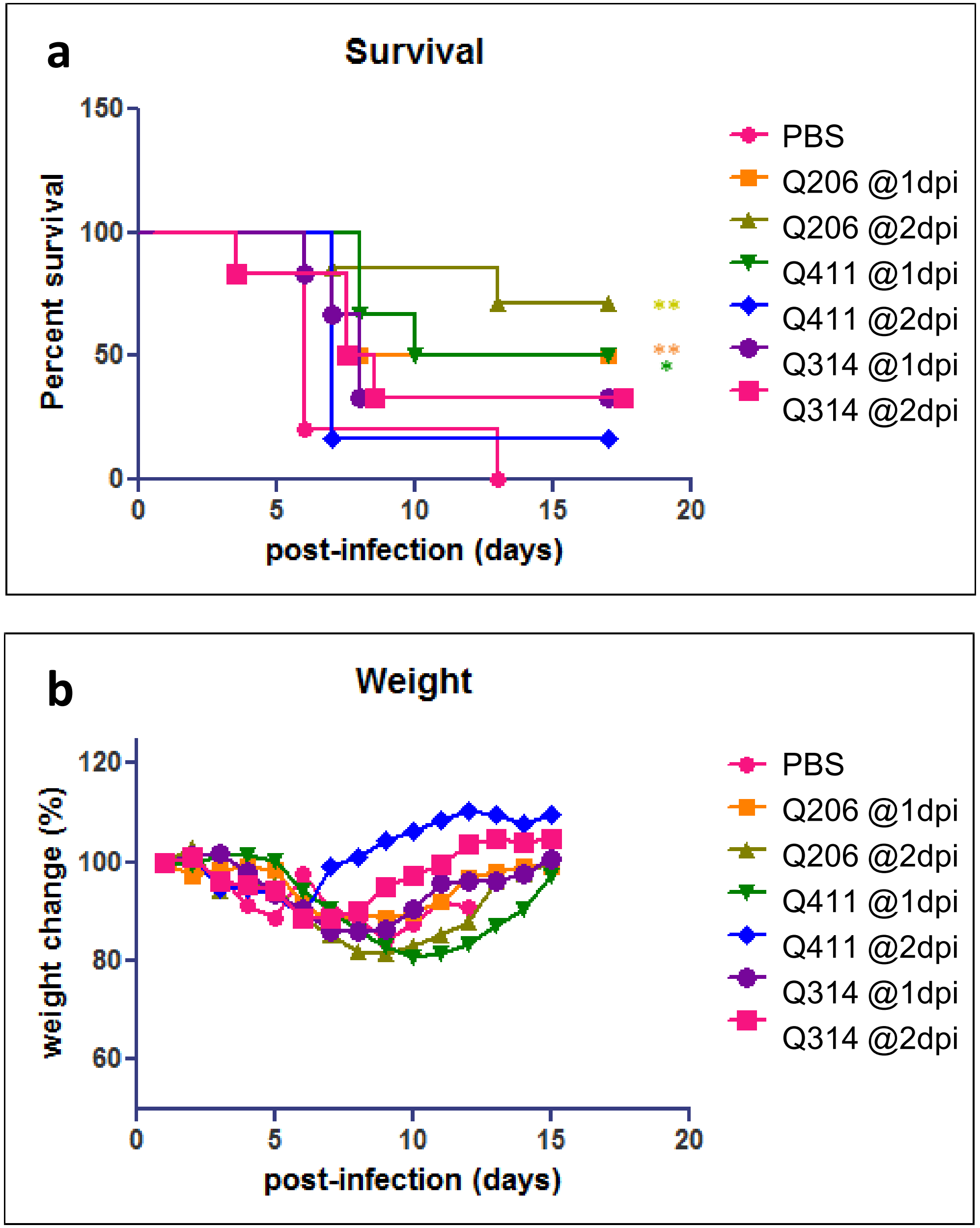

Supplementary Fig. 4

Supplementary Table 1. Statistics on negative-stain EM reconstruction

EM reconstruction statistics					
Sample	Pixel size(Å)	Number of micrographs	Number of boxed particles*	Number of particles for final refinement†	Resolution(Å)¶
GPdM/Fab Q206	1.68	116	19,860	1,101	27.5
GPdM/Fab Q314	1.68	301	40,250	4,127	30.2
GPdM/Fab Q411	1.68	216	53,846	6,136	25.2

* The numbers of overall particles boxed out using e2boxer.py program.
† The particles used to generate the final EM map using RELION auto-refine.
¶ Determined using golden standard Fourier shell correlation.
